# Quality of life and mental well‐being of adults with atopic dermatitis living in the UK

**DOI:** 10.1111/cea.14237

**Published:** 2022-10-06

**Authors:** Gurkiran Birdi, Michael Larkin, Ser‐Ling Chua, Rebecca C. Knibb

**Affiliations:** ^1^ School of Psychology, College of Health and Life Sciences Aston University Birmingham UK; ^2^ Department of Dermatology University Hospitals Birmingham NHS Foundation Trust Birmingham UK

**Keywords:** atopic dermatitis, mental health, psychodermatology, quality of life

**Key messages**

- Stress and Atopic Dermatitis severity significantly predict the quality of life in UK adults
- There are differences in Atopic Dermatitis severity and depression reported across different ethnicities.
- These factors need to be considered when supporting adults to manage their Atopic Dermatitis.

To the Editor,

Atopic dermatitis (AD) is a chronic inflammatory skin disease characterized by dry itchy skin and pruritus, with a 1‐year prevalence rate of 17% among adults in Europe.^1^ The UK has one of the highest prevalence rates of adult AD.^2^ Few studies have investigated the relationship between AD, quality of life (QoL) and mental well‐being such as stress, depression or anxiety in adults. Those that have reported inconsistent findings possibly due to small sample sizes or inappropriate measures were used.^3^ Socio‐demographic factors are also seldom taken into consideration despite significant differences between ethnic groups in severity and prevalence of AD.^4^ This study aims to overcome some of these issues using validated measures to explore the relationship between QoL, mental health, demographic and clinical variables in a large UK adult population reporting a clinical diagnosis of AD compared with a sample of adults with no AD.

This was a quantitative cross‐sectional study. It received NHS/HRA ethical approval (REC#: 18/NE/0228) and all participants gave informed consent before completing the measures. Adults (aged 18+ years) reporting a clinical diagnosis of AD were recruited from a dermatology clinic and in response to adverts on social media; a control group with no AD were recruited through social media. After giving consent all participants completed measures anonymously through an online survey. Socio‐demographic and AD‐specific information were collected. The *Skindex‐29* was completed by AD patients to measure AD‐specific QoL. The *Patient‐Oriented Eczema Measure (POEM)* was used to assess AD severity. The *Perceived Stress Scale (PSS14*), the *Hospital Anxiety and Depression Scale (HADS)* and the *WHOQoL‐BREF* (to measure general QoL) were completed by all participants. Due to multiple comparisons significance was set at *p* < 0.01. Sensitivity analysis showed that with alpha set at 0.01, the study had 80% power to detect small effect sizes for independent samples *t*‐tests (*d* = 0.30).

The survey was completed by 301 participants (56.5%) with AD and 232 healthy controls (43.5%). In the AD group, 43 participants (14.3%) were recruited through a dermatology clinic, and 258 participants (85.7%) responded to a social media advert. Overall, there were 390 (73.2%) females and 143 (26.8%) males; 378 (70.9%) were White, 139 (25.9%) Asian, and 17 (3.2%) were Black. The majority were aged between 25 and 39 (*N* = 262, 49.2%); 131 (24.6%) were aged between 18 and 24; 124 (23.3%) were aged between 40 and 60; and 14 (2.6%) were aged 61 and above. Almost half of the AD group reported suffering from allergies (*n* = 145, 48.2%), and/or asthma (*n* = 140, 46.5%). Just over half were prescribed topical corticosteroids (*n* = 162, 53.8%), 46.2% managed AD with emollients (*n* = 139), 3.3% were prescribed Dupilumab (*n* = 10), 11.3% topical calcineurin inhibitors (*n* = 34), 11% oral cortico‐steroids (*n* = 33) and 6.6% antibiotics (*n* = 20).

There were no significant differences between the AD group recruited from clinic or social media. There were significant differences between the AD and control groups for ethnicity and age (analysis not shown). Thus, all analyses comparing AD and control groups controlled for these variables. Those with AD reported lower general physical QoL (Mean = 12.10 SD = 2.84) compared with controls (Mean = 13.56 SD = 3.19) (*F*[1, 533] = 2.27, *p* < 0.001, η2*p*= 0.05); lower overall health (Mean = 2.83 SD = 1.14) compared with controls (Mean = 3.32 SD = 1.01) (*F*[1533] = 26.72, *p* < 0.001, η2*p* = 0.04); and greater anxiety (Mean = 11.17 SD = 4.09) compared with controls (Mean = 10.29 SD = 4.84) (*F*[1533] = 6.51, *p* = 0.01, η2*p* = 0.01). There were no other significant differences between groups.

In adults with AD, those diagnosed as a child had higher disease severity than those diagnosed as adults (*F*[2297] = 8.91, *p* < 0.001). Females reported higher AD severity (*t*[299] = −2.7, *p* = 0.007), and lower skin‐related QoL (*t*[299] = −2.81, *p* = 0.005) compared with males. Asians reported higher disease severity (*F*[2298] = 5.2, *p* = 0.006), compared with White and Black participants. Asians also reported lower AD‐related QoL (*F*[2298] = 7.04, *p* = 0.001) compared with White and Black participants (Table 1). Males reported higher depression levels (M = 9.45 SD = 3.23) compared with females (M = 8.35 SD = 4.01) (*t*[299] = 2.16, *p* = 0.002). White participants reported higher depression (M = 9.11 SD = 3.65) compared with Asian (M = 7.92 SD = 4.02) and Black (M = 7.80 SD = 4.23) participants, (*F*[2298] = 3.52, *p* = 0.001).

**TABLE 1:** Mean scores with standard deviations for differences between socio‐demographic groups, AD characteristics, QoL (measured by the Skindex‐29) and disease severity (measured by the POEM) in adults with AD

	QoL scores	
Ethnicity**		
White (*N* = 378)	3.21 (0.85)	
Asian (*N* = 138)	3.54 (0.84)	
Black (*N* = 17)	2.75 (1.11)	
Gender*		
Female (*N* = 390)	3.40 (0.86)	
Male (*N* = 143)	3.01 (0.86)	
	Yes [M (SD)]	No [M (SD)]
Antihistamines	3.60 (0.83)	3.20 (0.86)
Asthma	3.46 (0.81)	3.09 (0.87)
Calcineurin inhibitors**	3.76 (0.73)	3.26 (0.86)
Dupilumab*	3.91 (0.31)	3.30 (0.87)
Food allergy	3.59 (0.82)	3.26 (0.87)
Immunosuppressants*	3.67 (0.84)	3.27 (0.86)
Oral corticosteroids**	4.06 (0.62)	3.23 (0.85)
Sedative antihistamine*	3.80 (0.73)	3.28 (0.87)
Topical corticosteroids**	3.50 (0.90)	3.10 (0.77)
	Disease severity scores	
Ethnicity*		
White (*N* = 378)	15.52 (6.28)	
Asian (*N* = 138)	18.67 (8.46)	
Black (*N* = 17)	12.75 (8.32)	
Gender*		
Female (*N* = 390)	16.93 (6.82)	
Male (*N* = 143)	14.58 (6.25)	
Age of diagnosis**		
Child (*N* = 132)	17.82 (9.24)	
Adolescent (*N* = 81)	16.28 (6.84)	
Adult (*N* = 87)	16.19 (8.36)	
	Yes [M(SD)]	No [M(SD)]
Antihistamines	19.11 (6.66)	15.88 (6.66)
Asthma	18.02 (6.39)	13.91 (6.33)
Calcineurin inhibitors**	20.17 (5.01)	15.78 (6.77)
Children diagnosis	17.90 (6.8)	14.32 (6.26)
Topical corticosteroids*	18.88 (6.73)	13.88 (5.96)
Food allergy	18.32 (6.92)	15.39 (6.47)
Immunosuppressants*	19.60 (6.73)	15.86 (6.63)
Oral corticosteroids**	22.30 (4.75)	15.53 (6.58)
Sedative antihistamine**	21.95 (5.54)	15.86 (6.63)

**p < 0.001, *p < 0.01. Score range for QoL: 1 (never bothered me) – 5 (bothered me all the time); Score range for disease severity: 0 (clear or almost clear) – 28 (very severe).

There was a significant difference between severity of AD and QoL as measured by the Skindex‐29 (*F*[4296] = 59.23, *p* < 0.001). Those with clear AD reported significantly better QoL (M = 1.7, SD = 0.65) compared with mild (M = 2.4, SD = 0.7), moderate (M = 3.0, SD = 0.64), severe (M = 3.69, SD = 0.57) and very severe AD (M = 4.14, SD = 0.88). *Post‐hoc* analyses showed significant differences lay between all severity categories of the POEM. Adults reported significantly lower QoL if they were prescribed topical corticosteroids (*t*[295] = −4.05, *p* < 0.001), oral corticosteroids (*t*[296] = −5.40, *p* < 0.001), immunosuppressant medication (*t*[296] = −2.56, *p* = 0.01), Dupilumab (*t*[296] = −2.16, *p* = 0.004) were on topical calcineurin inhibitors (*t*[296] = −3.64, *p* = 0.001) or took sedating medication (*t*[296] = −2.64, *p* = 0.009), compared with those who were not (Table 1).

Adults reported significantly higher disease severity if they were prescribed topical corticosteroids (*t*[295] = −5.89, *p* < 0.01), oral corticosteroids (*t*[296] = −5.72, *p* < 0.001), immunosuppressant medication (*t*[296] = −3.052, *p* = 0.002), were on topical calcineurin inhibitors (*t*[296] = −3.65, *p* < 0.001) or took sedating medication (*t*[296] = −4.10, *p* < 0.001), compared with those who were not (Table 1).

Poorer general QoL, physical, psychological and social QoL, greater stress, anxiety, depression and greater disease severity were all significantly correlated with poorer AD‐specific QoL (correlations from 0.20–0.68). QoL and mental health predictors were entered into a hierarchical multiple regression model to explore predictors of AD‐specific QoL. Demographic and clinical factors were entered in the first step, anxiety, depression, stress, and QoL scores were entered in the second step, AD severity was added in the final step. All models were significant (data not shown). The final model (*F*[25,296] = 0.95, *p* < 0.001) explained 54% of the total variance in QoL (*R* ^2^ = 0.61, adj *R* ^2^ = 0.54). Physical QoL, social QoL, stress and AD severity were significantly associated with AD‐specific QoL. AD severity was the strongest predictor (Table 2 shows the final model with all predictors included).

**TABLE 2:** Predictors of atopic dermatitis specific quality of life in adults with AD (final step of the model)

	Unstandardized β	Standardized β	95% CI	
			Lower	Upper
Gender	0.02	0.01	−0.15	0.19
Topical corticosteroids	0.02	0.02	−0.08	0.11
Emollients	0.07	0.04	−0.08	0.22
Antihistamines	−0.04	−0.02	−0.32	0.23
Immunosuppressants	−0.19	−0.07	−0.49	0.11
Oral corticosteroids	0.20	0.07	−0.09	0.49
Sedating antihistamines	−0.19	−0.05	−0.62	0.23
Antibiotics	0.19	0.06	−0.19	0.58
Dupilumab	0.14	0.03	−0.31	0.59
Calcineurin inhibitors	0.09	0.03	−0.20	0.37
Diagnosis	0.10	0.06	−0.06	0.26
Allergy	−0.19	−0.11	−0.34	−0.03
Asthma	0.06	0.04	−0.10	0.22
Food allergy	0.11	0.06	−0.07	0.29
Ethnicity	0.09	0.05	−0.13	0.31
Age	0.20	0.11	0.01	0.38
Family history	0.06	0.03	−0.10	0.22
Stress	0.02	0.13*	0.00	0.03
Anxiety	0.02	0.09	−0.01	0.04
Depression	0.01	0.03	−0.02	0.04
Physical QoL	−0.06	−0.21**	−0.11	−0.02
Psychological QoL	−0.03	−0.10	−0.07	0.01
Social QoL	0.06	0.20**	0.02	0.10
Environmental QoL	−0.01	−0.05	−0.03	0.01
Overall QoL	0.07	0.09	−0.02	0.17
Health‐related QoL	0.04	0.05	−0.06	0.13
Disease severity	0.07	0.53***	0.06	0.08

*p < 0.05, **p < 0.01, ***p < 0.001.

This study is the first to investigate the association between AD, QoL and mental health in a large sample of UK adults. Adults with AD reported lower physical QoL and overall health compared with healthy controls. This could be due to the physical discomfort and pain that arises due to AD.^5^ No other differences were found between the AD and control groups for generic QoL in this study. This could suggest that AD has a more specific impact on certain areas of QoL that are connected to having a skin condition. Therefore, when comparisons with healthy groups are not needed, measuring QoL in those with AD using an AD‐specific QoL tool rather than a generic tool is recommended to help inform AD management. AD patients reported higher levels of anxiety compared with healthy controls. The incidence of psychiatric disorders among dermatological patients has been reported to be approximately 30%–40%^6^ and this should be considered when supporting adult patients with AD in managing their condition.

In our study poorer AD‐specific QoL was significantly related to poorer mental health, greater AD severity, medication use, age of diagnosis and presence of other allergies and asthma. AD severity was significantly associated with QoL after controlling for psychological factors, a finding consistent with a recent systematic review.^3^ Stress, general physical and social QoL were also significantly associated with AD‐specific QoL, after controlling for demographic and clinical variables. Those affected by AD are more restless in their sleep due to the itch associated with AD, wake more often, spend less time asleep and report daytime fatigue^7^ potentially explaining the relationships found here.

When examining demographic variables, increased AD severity and lower QoL was found in Asian participants compared with White and Black participants. The low numbers of Black participants in this study should be taken into consideration when interpreting these results, however this finding is reflected in previous literature where Asian patients present more severe skin symptoms such as lichenification (thickening of the skin) compared to White patients and are at higher risk for developing post‐inflammatory dyspigmentation.^8^ White participants scored higher for depression compared with BME groups, a finding not explored previously in those with AD. This may be due to White adults being more willing to report depressive symptoms compared with their minority‐ethnic counterparts but this finding warrants further investigation.

Females with AD in this study reported higher disease severity and lower QoL compared with males. Males in this study, however reported higher levels of depression compared with females. This is contrary to a gender‐specific association with AD and depression suggested by Timonen et al.^9^ where biological factors may explain higher rates of depression in females than males and so would also benefit from further study.

This is a large UK population‐based study of adults with AD, however some limitations should be taken into consideration. This study relies on the accuracy of self‐report for diagnosis of AD for those recruited outside of the dermatology clinic. The study had a high level of power to detect differences across the whole sample, but numbers in some of the comparison groups were small and so the study may not have been powered enough to detect differences across all sub‐samples analyzed and causality cannot be determined using this study design. Nevertheless, these data support the heavy mental health burden that AD places on patients and it is important for clinicians to recognize the impact that AD has on QoL and mental health when guiding patients who are managing this condition.

## AUTHOR CONTRIBUTIONS

All authors contributed to the study design and methods, GB collected data and conducted the analysis, GB and RCK wrote the manuscript, all authors edited and agreed the final version of the manuscript.

## FUNDING INFORMATION

This research did not receive any specific grant from funding agencies in the public, commercial, or not‐for‐profit sectors.

## CONFLICT OF INTEREST

The authors have no conflicts of interest in relation to this paper.

## Data Availability

The data that support the findings of this study are available on request from the corresponding author. The data are not publicly available due to privacy or ethical restrictions.
